# Awareness of, attitude toward, and willingness to participate in pay for performance programs among family physicians: a cross-sectional study

**DOI:** 10.1186/s12875-020-01118-9

**Published:** 2020-03-30

**Authors:** Chyi-Feng Jan, Meng-Chih Lee, Ching-Ming Chiu, Cheng-Kuo Huang, Shinn-Jang Hwang, Che-Jui Chang, Tai-Yuan Chiu

**Affiliations:** 1 Department of Family Medicine, National Taiwan University Hospital, College of Medicine, National Taiwan University, No.7, Chung-Shan South Road, 10002 Taipei, Taiwan; 2 Department of Family Medicine, Tainan Hospital, Ministry of Health and Welfare, Tainan, Taiwan; 3 Chinese Taipei (Taiwan) Association of Family Medicine, Taipei, Taiwan; 4 Department of Family Medicine, National Taiwan University Hospital, Yunlin Branch, Taipei, Yunlin Taiwan; 5 Keelung Medical Association, Keelung, Taiwan; 6 School of Medicine, National Yang-Ming University, Taipei, Taiwan; 7 Taipei Veterans General Hospital, Taipei, Taiwan

**Keywords:** Family physician, Pay-for-performance, Awareness, Attitude, Willingness

## Abstract

**Background:**

The National Health Insurance Administration of Taiwan has introduced several pay-for-performance programs to improve the quality of healthcare. This study aimed to provide government with evidence-based research findings to help primary care physicians to actively engage in pay-for-performance programs.

**Methods:**

We conducted a questionnaire survey among family physicians with age-stratified sampling from September 2016 to December 2017. The structured questionnaire consisted of items including the basic demographics of the surveyee and their awareness of and attitudes toward the strengths and/or weaknesses of the pay-for-performance programs, as well as their subjective norms, and the willingness to participate in the pay-for-performance programs. Univariate analysis and multivariate logistic regression analysis were performed to compare the differences between family physicians who participate in the pay-for-performance programs versus those who did not.

**Results:**

A total of 543 family physicians completed the questionnaire. Among family physicians who participated in the pay-for-performance programs, more had joined the Family Practice Integrated Care Project [Odds ratio (OR): 2.70; 95% Confidence interval (CI): 1.78 ~ 4.09], had a greater awareness of pay-for-performance programs (OR: 2.37; 95% CI: 1.50 ~ 3.83), and a less negative attitude to pay-for-performance programs (OR: 0.50; 95% CI: 0.31 ~ 0.80) after adjusting for age and gender. The major reasons for family physicians who decided to join the pay-for-performance programs included believing the programs help enhance the quality of healthcare (80.8%) and recognizing the benefit of saving health expenditure (63.4%). The causes of unwillingness to join in a pay-for-performance program among non-participants were increased load of administrative works (79.6%) and inadequate understanding of the contents of the pay-for-performance programs (62.9%).

**Conclusions:**

To better motivate family physicians into P4P participation, hosting effective training programs, developing a more transparent formula for assessing financial risk, providing sufficient budget for healthcare quality improvement, and designing a reasonable profit-sharing plan to promote collaboration between different levels of medical institutions are all imperative.

## Background

Pay for performance (P4P) programs have become an emerging quality improvement movement in healthcare systems worldwide [1, 2]. They aim at rendering payment for healthcare providers according to their value-based performance [3]. Healthcare facilities and providers are encouraged to observe essential guidelines to provide patients with comprehensive, continuous, and coordinative care through justified incentives for improving both the quality and value of healthcare. The major incentive factor for motivating P4P participation is to help physicians improve health outcome by following clinical guidelines and standard operational procedure [4, 5]. Quite a few P4P programs have been implemented in Taiwan and all over the world in response to the healthcare demands of our increasingly aging global village [6–9]. In terms of manpower, primary care physicians, especially those specializing in family medicine, form the cornerstone of P4P programs [10, 11].

Taiwan has launched a universal healthcare coverage plan called “National Health Insurance Plan” since 1995. Starting from 2001, the National Health Insurance Administration (NHIA) has selected several common diseases to be handled under “P4P” design with the hope to enhance healthcare quality and efficiency. Diseases currently covered under P4P programs include: diabetes mellitus, asthma, breast cancer, cervical cancer, hypertension, tuberculosis, chronic hepatitis B and C, schizophrenia, early stage chronic kidney disease, comprehensive maternal care for pregnant women, early treatment for development retardation, chronic kidney disease, chronic obstructive lung disease, and others [12]. Moreover, NHIA also has implemented the Family Practice Integrated Care Project (FPICP) since March 2003 to promote community health care group (CHCG)-based practice in the primary care sector, striving to recruit more primary care practitioners to join force in establishing primary community care networks (PCCNs) island-wide in Taiwan [13]. Above all, NHIA P4P programs intend to provide reasonable incentives such as expense reimbursement to drive healthcare providers to deliver holistic and quality care.

All stakeholders, including health plans, physicians, and patients, would benefit from health plans collaborating on their P4P efforts to maximize physician participation [14]. Foels T et al. also suggest that the success of P4P programs for diabetes care is attributed to the engagement of physicians, actionable reports, office-based education, written action plans, and alignment with internal disease management [15]. Cross DA et al. recommend delivering best care through external learning opportunities, and fostering intrinsic motivation to pursue transformational improvements in chronic disease patient care [16]. Girault A et al. suggest increasing clarity in P4P program rules and awareness among hospital staff potential challenges, as well as incentivizing time-consuming quality care to P4P program efficacy [17]. The numbers of P4P programs primary care physicians’ participants in Taiwan are growing but still limited. Therefore, to understand the willingness of family physicians to participate in P4P programs, this study aimed to investigate the following four issues: first, the current status of P4P participation among family physicians in Taiwan; second, the relationship between the characteristics of family physicians and their willingness to participate in P4P programs with a focus on the differences between junior doctors and senior doctors; third, the relationship between family physicians’ awareness of P4P programs, their subjective norms, and their P4P participation willingness; and the last, the determining factors affecting family physicians’ willingness to participate in P4P programs.

## Methods

### Study design

**Fig. 1:**
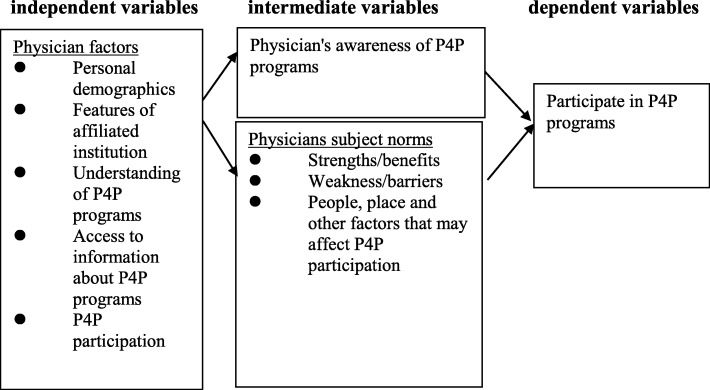
The conceptual framework of the study. P4P: Pay for performance

The conceptual architecture of this study was modified based on Rosenstock’s Health Belief Model after adding the factors of research interest. (Fig. 1) [18] The independent variables included physician demographic data (physician traits), and intermediate factors covered physician’s awareness scores on P4P programs, their attitude towards P4P programs, and their subjective norms. The dependent variable or the outcome was “Whether family physicians participate in P4P programs.”

### The characteristics of participants

The study conducted a survey during the period from September 2017 to December 2017 on TAFM (the Taiwan Association of Family Medicine) board certified family physicians based on age stratified sampling. At the time of this study, there are 5186 Family Medicine board-certified members in TAFM. As this study would like to explore the active members’ opinion for practice, 725 members aged over 70 years were excluded. The remaining 4461 certified family physicians were placed in four 10-year age groups from 30 to 70 years, with an equal number of participants in each age group selected by random sampling based on their TAFM certificated numbers so as to ensure sample representativeness. We predicted effective sample size of 250 to 500 following the rule of 5 to 10 times the number of questions in the questionnaire, so we aimed to gather 500–600 participants finally [19, 20]. As the response rate of questionnaire survey is estimated 20~25% for doctors in Taiwan, we draw 2000 physicians for final mailing list [21]. We sent the paper questionnaire by mail and electronic version by Email two times through the assistance of TAFM staffs to increase the response rate. We also provided 250 NTD (8 USD) gift card of convenience store for every responder. The study participants were divided into two groups based on duration of practice since first-year residence. Participants with a duration of practice less than 7 years were regarded as junior doctors, and those over 7 years as senior doctors.

### Method

A structured questionnaire was developed, covering items concerning the basic demographics of the study participants, their awareness of and attitudes toward the strengths (benefits) and weaknesses (barriers) of P4P programs, their subjective norms, and P4P participation willingness. The characteristics of surveyee included: age, gender, level of affiliated medical institution, CHCG enrollment, and duration of practice. Ten items of the questionnaire aimed at assessing awareness of P4P programs with a respondent scoring either 0 or 1 point for each item. Study participants scoring 7 points or higher were deemed to have a better understanding of P4P programs using last one quantile as baseline. For measuring attitude, a 5-point Likert-type scale based on the degree of agreement and importance was used for understanding the opinions of the study participants about the strengths/benefits and weaknesses/barriers of P4P programs. A higher score, defined as 18 points or over in a total of 30 points, indicated a more positive attitude toward P4P programs assuming the last one quantile as baseline. The subjective norms of the surveyed family physicians, that is, the people, places, events, or other factors that might affect P4P participation, were also measured by a 5-point Likert-type scale whose total score read 20 points, and a score reaching or exceeding 12 points was regarded as relatively higher subjective norms assuming the last one quantile as baseline.. With respect to the measurement of P4P participation willingness, another 5-point Likert-type scale was used to examine positive as well as negative affecting factors with 4 or 5 points defined as a higher score indicating greater willingness providing the last one quantile as baseline.

Five experts were invited to assess the validity of the questionnaire content, paying special attention to whether the content was relevant and the text clear. In terms of surface validity, the questionnaire was pre-tested by 10 resident medical doctors to facilitate correction of the textual language and safeguard its clarity. We performed the reliability and validity testing for the questionnaire. The overall content validity index was 0.98 regarding importance as well as adequacy. The confirmatory factor analysis of the eight-factor model without cross-loadings shows that the Cronbach’s alpha estimates for the composite measures range from 0.71 to 0.86, indicating acceptable internal consistency. The values of the composite reliability for the composite measures range from 0.69 to 0.86. For the convergent validity, the estimates of the factor loadings of the single-item measures into the composites are all significant at *p* < 0.0001. The correlations among the eight composites range from − 0.45 to 0.86.

The study was approved by the Institutional Review Board of Clinical Ethics at National Taiwan University Hospital (NO. 201702014RIND) and funded by grants from the Ministry of Scientific Technology, Taiwan (MOST 106–2314-B-002 -110) and the TAFM.

### Statistical analysis

Univariate analysis was used to calculate the frequency for each item, including demographics, awareness, attitudes, subjective norms, and willingness. The differences between the two groups of P4P-participating and non-participating family physicians were examined by t test for continuous variables, such as age, knowledge, and subjective norms. Chi-square test was performed to compare categorical variables, including, gender, level of affiliated medical institution, CHCG participation, and subjective norms, etc. Logistic regression analysis was conducted to assess the above variables’ effects on P4P participation willingness. We analyzed the data using SAS/STAT® software and SPSS 20 software for statistics. Statistical significance was set a *p*-value < 0.05.

## Results

Of the 2000 sampled family physicians, 543 completed the questionnaire, resulting in a response rate of 27.2%. The mean age of the respondents was 48.1 ± 12.6 years old. 49.0% of them had joined at least one P4P program, and the top three higher P4P programs participation are diabetes (34.4%), early stage CKD (30.0%), as well as asthma (12.5%). 67.7% were primary health care providers in community settings, and 78.3% worked in private clinics. Most (82.4%) of them had been family physicians for at least 7 years since first-year residence. P4P participants and non-participants showed no difference in gender, mean age, work area, and duration of practice. Working in public medical institutions or hospitals, participating in the FPICP, and sufficient self-reported understanding of P4P programs appeared to indicate a greater willingness to participate in P4P programs. (*p* < 0.001) (Table 1).

**Table 1:** The characteristics of surveyed family physicians (pay-for-performance programs participants vs. non-participants)

Items		Participants (*n* = 266)^a^ N (%)	Non-participants (*n* = 277)^a^ N (%)	*p*-value
Gender	Male	206 (48.6)	218 (51.4)	0.70
	Female	60 (50.4)	59 (49.6)	
Mean age (+ − SD)		47.6 ± 12.8	48.5 ± 12.5	0.42
Location of affiliated medical institution defined by NHIA	North	149 (47.6)	164 (52.4)	0.55
	others	112 (50.2)	111 (49.8)	
Level of affiliated medical institution	primary care clinic or public health center	158 (43.1)	209 (57.0)	< 0.001
	Hospitals	108 (61.7)	67 (38.3)	
Management style	private	193 (45.8)	228 (54.2)	0.002
	public	72 (61.5)	45 (38.5)	
Participation in community health care group	Participant	145 (59.7)	98 (40.3)	< 0.001
	Non-participants	111 (39.1)	173 (60.9)	
Duration of practice	less than 7 years	45 (47.3)	50 (52.6)	0.73
	at least 7 years	219 (49.3)	225 (50.7)	
Self-reported understanding of P4P program	not enough	130 (37.6)	216 (62.4)	< 0.001
	enough	132 (71.4)	53 (28.7)	

*NHIA* National Health Insurance Administration, Taiwan

*P4P* Pay for performance

^a^Some data are missing

Table 2 shows the awareness of and attitude toward P4P programs between participants and non-participants. The P4P participation rate of the surveyed family physicians marked with a higher awareness score was higher than that of those reporting a lower awareness score (54.4% vs. 33.8%; *p* < 0.001). Regarding attitude towards P4P programs, for strengths and benefits, the surveyee showing higher scores in either agreement or importance were more likely to participate in P4P programs. (52.1% vs. 31.3, and 53.2% vs. 24.4%). For weaknesses and barriers, those reporting lower scores in agreement and importance were more likely to participate in P4P programs. (62.6% vs. 45.1 and 58.6% vs. 49.4%; *p* < 0.01).

**Table 2:** The awareness of and attitudes toward pay-for-performance programs among surveyed family physicians^a^

Items			Participants(*n* = 266)^a^ N (%)	Non-participants (*n* = 277)^a^ N (%)	*P* value
**Awareness score**					
7 points and higher (one item one point; total 10 items)		Yes	218 (54.4)	183 (45.6)	< 0.001
		No	48 (33.8)	94 (66.2)	
**Attitude towards P4P programs**					
Strengths/ Benefits	agreement	low	20 (31.3)	44 (68.8)	0.001
		high	244 (52.1)	224 (47.8)	
	importance	low	11 (24.4)	34 (75.6)	< 0.001
		high	235 (53.2)	207 (46.8)	
Weaknesses/Barriers	agreement	low	87 (62.6)	52 (37.4)	< 0.001
		high	178 (45.1)	217 (54.9)	
	importance	low	41 (58.6)	29 (41.4)	0.16
		high	206 (49.4)	211 (50.6)	

^a^Some data are missing

^b^pay-for-performance programs participants vs. non-participants

In terms of subjective norms that might affect P4P participation decision, NHIA (79.0%), peers/colleagues (67.4%), public health bureaus (61.1%) and senior doctors (56.4%) appeared to be the top four affecting factors. **(**Table 3**)** Regarding the surveyee’ perceptions about P4P programs that might affect their participation willingness, more than half of them who agreed on issues like the benefits of P4P programs in promoting healthcare quality and reducing NHI expenditure showed greater willingness to participate in the P4P programs. As for perceptions that might dampen participation willingness, P4P-participating respondents outnumbered their non-participating counterparts in registering disagreement on the following items: “It is not easy for P4P programs to demonstrate the healthcare quality they claims to improve,” (28.4% vs. 14.2%) “Participation in P4P programs involves steep financial risk,” (29.2% vs. 16.7%), “It is not an easy task understanding P4P programs,” (13.9% vs. 5.8%) and “Current reward incentives and quality and other related indicators are not satisfying enough to induce my P4P participation willingness” (19.3% vs. 9.5%; (*p* < 0.01). **(**Table 4**).**

**Table 3:** Subjective norms among surveyed family physicians (pay-for-performance programs participants vs. non-participants)

Items	Participants (*n* = 266)^a^ N (%)	Non-participants (*n* = 277) ^a^ N (%)	*P* value
National Health Insurance Administration (NHIA)			0.03*
Unlikely to affect	16(6.0)	26(9.5)	
Neutral or no opinion	26(9.8)	43 (15.6)	
Likely to affect	223 (84.2)	206 (74.9)	
Public Health Bureaus			0.04*
Unlikely to affect	40 (15.2)	43 (15.8)	
Neutral or no opinion	48 (18.2)	73 (26.8)	
Likely to affect	176 (66.7)	156 (57.4)	
Peers and colleagues			0.36
Unlikely to affect	32 (12.1)	30 (11.0)	
Neutral or no opinion	49 (18.5)	64 (23.5)	
Likely to affect	184 (69.4)	178 (65.4)	
Senior doctors			0.44
Unlikely to affect	45 (17.1)	37 (13.7)	
Neutral or no opinion	76 (28.9)	75 (27.7)	
Likely to affect	142 (54.0)	159 (58.7)	
Nurses			0.20
Unlikely to affect	60 (22.8)	59 (21.8)	
Neutral or no opinion	82 (31.2)	104 (38.4)	
Likely to affect	121 (46.0)	108 (39.9)	
Other medical professionals			0.55
Unlikely to affect	67 (25.4)	69 (25.4)	
Neutral or no opinion	93 (35.2)	107 (39.3)	
Likely to affect	104 (39.4)	96 (35.3)	
Friends			0.37
Unlikely to affect	128 (48.7)	129 (47.4)	
Neutral or no opinion	100 (38.0)	95 (34.9)	
Likely to affect	35 (13.3)	48 (17.6)	
Patients and their families			0.60
Unlikely to affect	18 (8.3)	107 (33.3)	
Neutral or no opinion	74 (34.1)	75 (23.4)	
Likely to affect	125 (57.6)	139 (43.3)	
Family			0.01*
Unlikely to affect	139 (52.9)	107 (39.6)	
Neutral or no opinion	79 (30.0)	99 (36.7)	
Likely to affect	45 (17.1)	64 (23.7)	

^a^Some data are missing

*p<0.05

**Table 4:** Perceptions about pay-for-performance programs of surveyed family physicians that may affect participation willingness*

Items		Participants (*n* = 266)^a^ N (%)	Non-participants (*n* = 277)^a^ N (%)	*P* value
P4P programs help enhance healthcare quality	Agree	215 (80.8)	177 (64.1)	< 0.001*
	Neutral or no opinion	37 (13.9)	70 (25.4)	
	Disagree	14 (05.3)	29 (10.5)	
P4P programs help reduce NHI expenditure	Agree	168 (63.4)	140 (50.7)	0.002*
	Neutral or no opinion	75 (28.3)	88 (31.9)	
	Disagree	22 (8.3)	48 (17.4)	
P4P programs helps increase income.	Agree	111 (42.0)	96 (34.9)	0.08
	Neutral or no opinion	109 (41.3)	114 (41.5)	
	Disagree	44 (16.7)	65 (23.6)	
P4P programs help decrease the dependence of fee-for-service model on quantity of care, thereby improving physicians’ quality of life	Agree	134 (51.0)	125 (45.6)	0.44
	Neutral or no opinion	89 (33.8)	100 (36.5)	
	Disagree	40 (15.2)	49 (17.9)	
It is not easy for P4P programs to demonstrate the healthcare quality they claims to improve	Agree	86 (33.0)	118 (42.9)	< 0.001*
	Neutral or no opinion	101 (38.7)	118 (42.9)	
	Disagree	74 (28.4)	39 (14.2)	
Participation in P4P programs involves steep financial risk	Agree	81 (31.2)	93 (33.8)	< 0.001*
	Neutral or no opinion	103 (39.6)	136 (49.5)	
	Disagree	76 (29.2)	46 (16.7)	
Participation in P4P programs increases workload	Agree	206 (79.2)	218 (79.6)	0.19
	Neutral or no opinion	35 (13.5)	45 (16.4)	
	Disagree	19(7.3)	11(4.0)	
It is not an easy task understanding P4P programs	Agree	160 (61.8)	173 (62.9)	0.004*
	Neutral or no opinion	63 (24.3)	86 (31.3)	
	Disagree	36 (13.9)	16(5.8)	
Participation in P4P programs may increase the risk of medical disputes	Agree	35 (13.5)	63 (22.9)	< 0.001*
	Neutral or no opinion	101 (38.8)	143 (52.0)	
	Disagree	124 (47.7)	69 (25.1)	
Current reward incentives and quality and other related indicators are not satisfying enough to induce my P4P participation willingness	Agree	102 (39.4)	133 (48.7)	0.003*
	Neutral or no opinion	107 (41.3)	114 (41.8)	
	Disagree	50 (19.3)	26(9.5)	

*P4P* Pay for performance

^a^Some data are missing

*pay-for-performance participants vs. non-participants

As shown in Table 5, over 80% of the surveyed family physicians agreed on all of the four suggested measures for increasing P4P participation willingness, including hosting training programs to facilitate better understanding about P4P programs, developing a more transparent formula for assessing financial risk, providing adequate budget to improve healthcare quality, and drafting a reasonable profit-sharing plan to expedite collaboration between different levels of medical institutions (*p* < 0.01).

**Table 5:** Opinions of surveyed family physicians on potential measures for increasing pay-for-performance participation willingness*

Items		Participants(*n* = 266)^a^ N (%)	Non-participants(*n* = 277)^a^ N (%)	*P* value
Hosting training programs to facilitate better understanding about P4P programs	Agree	236 (88.7)	199 (71.8)	< 0.001*
	Neutral or no opinion	22(8.3)	69 (24.9)	
	Disagree	8(3.0)	9(3.2)	
Developing more transparent formula for assessing financial risk	Agree	244 (92.1)	218 (78.7)	< 0.001*
	Neutral or no opinion	19(7.2)	47 (17.0)	
	Disagree	2(0.8)	12(4.3)	
Providing adequate budget to improve healthcare quality	Agree	253 (95.5)	230 (83.3)	< 0.001*
	Neutral or no opinion	9(3.4)	40 (14.5)	
	Disagree	3(1.1)	6(2.2)	
Drafting reasonable profits-sharing plan to expedite collaboration between different levels of medical institutions	Agree	247 (93.6)	226 (81.6)	< 0.001*
	Neutral or no opinion	15(5.7)	43 (15.5)	
	Disagree	2(0.8)	8(2.9)	

*P4P* Pay for performance

^a^Some data are missing

*pay-for performance participants vs. non-participants

Results of univariate logistic regression analysis indicated that the surveyed family physicians marked with a better understanding about P4P programs, higher subjective norms, a more positive attitude toward P4P programs, and FPICP participation were respectively 2.65 times (95%C.I.:1.72~ 4.09), 2.60 times (95%C.I.:1.44~ 4.68), 2.37 times (95%C.I.:1.33~ 4.25), and 2.27 times (95%C.I.:1.56~ 3.31) more likely to participate in P4P programs. On the contrary, those showing a more negative attitude toward P4P programs were 55% (95%C.I.: 29%~ 71%) less likely to participate in P4P programs. After adjustment for the age and gender, the most striking factors affecting P4P participation willingness were FPICP participation (2.70 times, 95%C.I.:1.78~ 4.09), better understanding about P4P programs (2.39 times, 95%C.I.:1.50~ 3.83), as well as a negative attitude toward P4P programs (0.50 times, 95%C.I.:0.31~ 0.8). **(**Table 6**).**

**Table 6:** Logistic regression analysis to see the actual participating in pay-for-performance programs

Items		Odds Ratio (95% C.I.)		Adjusted Odds Ratio (95% C.I.)	
Age	Continuous variables	0.99	(0.98~1.00)	0.98	(0.96~1.00)
Gender	Male /female (1)	0.97	(0.62~1.52)	1.15	(0.68~1.92)
Better understanding about P4P programs	Answering 7 or more items/answering less than 7 items out of a total of 10 items (1)	2.65	(1.72~4.09)^*^	2.39	(1.50~3.83)^*^
Positive attitude toward P4P programs	Scoring 18 points or higher/scoring lower than 18 points out of a total score of 30 points (1)	2.37	(1.33~4.25)^*^	1.12	(0.55~2.30)
Negative attitude toward P4P programs	Scoring 18 points or higher/scoring lower than 18 points out of a total score of 30 points (1)	0.45	(0.29~0.71)^*^	0.50	(0.31~0.80)^*^
Higher subjective norms	Scoring 12 points or higher/scoring lower than 12 points out of a total score of 20 points (1)	2.60	(1.44~4.68)^*^	1.99	(0.97~4.11)
FPICP participation	Yes/no (1)	2.27	(1.56~3.31)^*^	2.70	(1.78~4.09)^*^

*P4P* Pay for performance

*FPICP* Family Practice Integrated Care Project

*C.I.* Confidence Interval

Adjusted odds ratio: adjusted age, gender

*p < 0.05

## Discussion

To the best of our knowledge, this study is the first one aiming to understand the current status of P4P participation among family physicians in Taiwan. It provides public health government with evidence-based research findings to help primary care physicians to actively engage in P4P programs. The main results identified the following major affecting factors for family physicians to join the P4P programs: better awareness and understanding of P4P programs, currently FPICP participants, and a less negative attitude toward P4P programs.

Consistent with previously reported results, our study indicates that better awareness of P4P programs is associated with greater willingness to participate in P4P programs [11, 22]. However, the P4P programs participation rate is still low(< 35%) from the result of this study. Fisher ES found that the major weaknesses and barriers lie in the modest magnitude of incentives from resistance or indifference among physicians and the actual realization of quality improvement [23]. Our study indicates that neither age nor duration of practice are significant factors affecting P4P participation willingness, but the surveyed family physicians showing a more positive attitude toward P4P programs through recognition of more benefits and less barriers are more willing to participate in P4P programs. It is worth noting that more than three-fourths of the surveyee still disclose a low understanding about P4P programs. As the main workforce of P4P programs comes from primary care physicians, the results of our study can persuade the government health authorities to promote better understanding of P4P programs among family physicians.

Table 3 points out that the surveyee likely to be affected by NHIA and public health bureaus but unlikely to be affected by their families seemed more inclined to participate in P4P programs. They seem to concern more about issues related to payment, governance, and peers influence. Berendsen AJ et al. found that ‘developing personal relationships’ and ‘gaining mutual respect’ appear to dominate as the motivational factors to develop new collaborative care model [11]. Nevertheless, issues concerning physicians’ decreased clinical autonomy and loss of professionalism need to be addressed with considerable care and sensitivity while implementing P4P programs [24–26]. A systematic review conducted by Mendelson et al. in 2017 emphasized that value-based payments are likely to improve quality of care and reduce costs [27]. In other words, we need more evidence-based studies on the long term outcome of P4P programs to convince primary care physicians to participate in P4P programs for quality improvement in healthcare. Using diabetic care as an example, in Taiwan, most related studies show that diabetes P4P programs result in favorable outcome in process (examining HbA1C, LDL), patient outcome (decreasing mortality, nephropathy, and cost-effectiveness analyses) [28–31]. The other limitations of P4P initiatives for physicians in designing and implementing P4P programs include cost consideration, standardized reward systems, rewards allocation among team members, and barriers to organizational changes aiming at influencing the behaviors of individual physicians [32, 33]. Above all, the importance of effective and efficient delivery of accurate information to primary care physicians cannot be overemphasized as it certainly helps enhance their willingness to participate in P4P programs. The payment system needs to be revamped in a way that render physicians more willing to invest in the value-based payment model for quality healthcare. And less administrative procedures and paperwork should be taken a priority for lowering the barrier against P4P participation among family physicians [8, 34].

As to limitations of this study, in spite of our effort to ensure sample representativeness by doing our best to perform systemic age-stratified random sampling, our cross-sectional questionnaire survey relies mainly on the NHIA data of family physicians. Moreover, due to our exclusive focus on family physicians, the findings of the study may not be applicable to physicians of other medical specialties. Further studies are needed to explore whether primary care physicians of other specialties demonstrate similar attitudes and concerns related to P4P participation. It should also be noted that, because of limited time, our study did not incorporate all the constructs from providers’ perspectives, such as clinical relevance, cooperation, unintended consequences, control, financial salience, and impact [35].

## Conclusions

Our finding suggests that P4P program design, and the experience of P4P participants are key issues demanding careful consideration in facilitating sustainable development of P4P programs. To better motivate family physicians into P4P participation, this study recommends increasing physicians’ awareness and understanding of P4P programs, providing technical and educational support, reducing administrative burden, forging a cooperative relationship with other medical facilities or healthcare providers, developing more accurate quality measures, and minimizing unintended consequences.

## Data Availability

The datasets used and/or analysed during the current study are available from the corresponding author on reasonable request.

## Abbreviations

CHCG: Community Health Care Group
FPICP: Family Practice Integrated Care Project
LDL: Low density lipoprotein
NHIA: National Health Insurance Administration
P4P: Pay for performance
PCCN: Primary Community Care Network
TAFM: Taiwan Society of Family Medicine
